# Change in cervical length after arrested preterm labor and risk of preterm birth

**DOI:** 10.1002/uog.23653

**Published:** 2021-11-01

**Authors:** K. N. Rennert, S. H. Breuking, E. Schuit, M. N. Bekker, M. Woiski, M. A. de Boer, M. Sueters, H. C. J. Scheepers, M. T. M. Franssen, E. Pajkrt, B. W. J. Mol, M. Kok, F. J. R. Hermans

**Affiliations:** ^1^ Faculty of Medicine, Vrije Universiteit Amsterdam Amsterdam The Netherlands; ^2^ Department of Obstetrics and Gynaecology, Amsterdam Reproduction & Development Research Institute, Amsterdam UMC University of Amsterdam Amsterdam The Netherlands; ^3^ Julius Center for Health Sciences and Primary Care, University Medical Center Utrecht Utrecht University Utrecht The Netherlands; ^4^ Department of Obstetrics and Gynaecology University Medical Center Utrecht Utrecht The Netherlands; ^5^ Department of Obstetrics and Gynaecology Radboud University Nijmegen Medical Center Nijmegen The Netherlands; ^6^ Department of Obstetrics and Gynaecology Amsterdam Reproduction & Development Research Institute, Amsterdam UMC, Vrije Universiteit Amsterdam The Netherlands; ^7^ Department of Obstetrics and Gynaecology Leiden University Medical Center Leiden The Netherlands; ^8^ Department of Obstetrics and Gynaecology Maastricht University Medical Center Maastricht The Netherlands; ^9^ Department of Obstetrics University Medical Center Groningen Groningen The Netherlands; ^10^ Department of Obstetrics and Gynaecology, School of Medicine Monash University Melbourne Victoria Australia; ^11^ Aberdeen Centre for Women's Health Research University of Aberdeen Aberdeen UK

**Keywords:** arrested preterm labor, cervical length, change in cervical length, preterm birth, threatened preterm labor

## Abstract

**Objective:**

To assess the association between preterm birth and cervical length after arrested preterm labor in high‐risk pregnant women.

**Methods:**

In this *post‐hoc* analysis of a randomized clinical trial, transvaginal cervical length was measured in women whose contractions had ceased 48 h after admission for threatened preterm labor. At admission, women were defined as having a high risk of preterm birth based on a cervical length of < 15 mm or a cervical length of 15–30 mm with a positive fetal fibronectin test. Logistic regression analysis was used to investigate the association of cervical length measured at least 48 h after admission and of the change in cervical length between admission and at least 48 h later, with preterm birth before 34 weeks' gestation and delivery within 7 days after admission.

**Results:**

A total of 164 women were included in the analysis. Women whose cervical length increased between admission for threatened preterm labor and 48 h later (32%; *n* = 53) were found to have a lower risk of preterm birth before 34 weeks compared with women whose cervical length did not change (adjusted odds ratio (aOR), 0.24 (95% CI, 0.09–0.69)). The risk in women with a decrease in cervical length between the two timepoints was not different from that in women with no change in cervical length (aOR, 1.45 (95% CI, 0.62–3.41)). Moreover, greater absolute cervical length after 48 h was associated with a lower risk of preterm birth before 34 weeks (aOR, 0.90 (95% CI, 0.84–0.96)) and delivery within 7 days after admission (aOR, 0.91 (95% CI, 0.82–1.02)). Sensitivity analysis in women randomized to receive no intervention showed comparable results.

**Conclusion:**

Our study suggests that the risk of preterm birth before 34 weeks is lower in women whose cervical length increases between admission for threatened preterm labor and at least 48 h later when contractions had ceased compared with women in whom cervical length does not change or decreases. © 2021 The Authors. Ultrasound in Obstetrics & Gynecology published by John Wiley & Sons Ltd on behalf of International Society of Ultrasound in Obstetrics and Gynecology.

**CONTRIBUTION**

**What are the novel findings of this work?**

The risk of preterm birth before 34 weeks' gestation is lower in women with an increase in cervical length between admission for threatened preterm labor and at least 48 h later when contractions have ceased.

**What are the clinical implications of this work?**

By measuring cervical length both at admission for threatened preterm labor and after 48 h when contractions have ceased, physicians may be able to identify more precisely women at low risk for preterm birth who can be returned to routine antenatal care.

## INTRODUCTION

Preterm birth is one of the leading causes of neonatal morbidity and mortality worldwide, being responsible for 40% of all deaths in children under the age of five, and it accounts for various short‐ and long‐term neonatal complications^1^, ^2^, ^3^, ^4^. As preterm birth continues to place a substantial burden on health services, identifying women at high risk is essential.

A special group comprising 9% of pregnant women presents with threatened preterm labor, which often requires hospital admission^5^. Promising markers for risk stratification of preterm birth in these women are transvaginal cervical‐length measurement and fetal fibronectin testing. Cervical length has an inverse relationship with the risk of preterm birth in symptomatic women, and dynamic shortening of cervical length is also associated with preterm birth^6^, ^7^, ^8^. Similarly, elevated fetal fibronectin levels have been linked to an increased risk of preterm birth in women with threatened preterm labor^9^, ^10^, ^11^.

It remains a clinical challenge to differentiate between high and low risk of preterm birth in women presenting with threatened preterm labor, as over half of these women eventually deliver at term^12^. This is especially the case for women who are defined as being at high risk of preterm birth based on cervical length and fetal fibronectin status at admission, but who have arrested preterm labor after 48 h of tocolysis^10^, ^13^. To date, little is known about the value of an additional transvaginal cervical‐length measurement following an episode of threatened preterm labor after contractions have ceased. This could aid physicians to identify women with a persistently increased risk of preterm birth who should remain under hospital care and those with a low risk who can be returned to routine antenatal care.

The aim of this study was to investigate the association of transvaginal cervical length at least 48 h after arrested preterm labor, both as a single measurement and as a change relative to the measurement at admission, with preterm birth in high‐risk women.

## METHODS

This study was a *post‐hoc* analysis of a randomized controlled trial (RCT), Apostel‐VI, the design and primary results of which have been published elsewhere^14^, ^15^. Briefly, 129 women with a singleton pregnancy and 35 women with a twin pregnancy between 24 + 0 and 34 + 6 weeks' gestation who did not deliver after an episode of threatened preterm labor were included. The women were triaged as having a high risk of preterm birth and were randomized into placement of a cervical pessary and no treatment (routine care) after contractions had ceased. At admission, all women were triaged for the risk of preterm birth based on a prognostic model. This model defined high risk of preterm birth as a cervical length of < 15 mm or a cervical length between 15 and 30 mm with a positive fetal fibronectin test. Women received corticosteroids and tocolysis according to the national Dutch guideline on threatened preterm labor^16^.

Cervical length was measured transvaginally at admission (CL1) and prior to randomization (CL2). Women were randomized between 48 and 120 h after admission when contractions had ceased and tocolysis had been discontinued. Measurements were obtained by trained midwives or gynecologists.

Women with ruptured membranes, cervical dilatation of ≥ 3 cm or residual cervical length that made it impossible to place a pessary were excluded. The cervical pessary remained in place until 36 + 0 weeks or until delivery.

The primary results of the RCT showed that placement of a cervical pessary neither reduced the rate of preterm birth nor had an effect on the rate of adverse perinatal outcome.

### Statistical analysis

Baseline variables were summarized using descriptive statistics. The characteristic of interest was cervical length at least 48 h following admission for threatened preterm labor when contractions had ceased. This variable was analyzed as a single measurement (CL2) and as change in cervical length compared with the measurement at admission (CL1) on a continuous scale (Δ_CL_ = CL2 – CL1). In addition, Δ_CL_ was categorized into ‘decrease’, ‘no change’ or ‘increase’ in cervical length. The margin of error around no change in cervical length was set at ± 2 mm^17^, ^18^. The outcomes of interest were preterm birth before 34 weeks and delivery within 7 days after admission for threatened preterm labor. Linearity between the determinants (CL2 and Δ_CL_) and outcome variables (preterm birth before 34 weeks and delivery within 7 days after admission) was assessed with restricted cubic splines. All associations were linear.

Logistic regression analysis was performed to produce association models with CL2 and Δ_CL_ as determinants on a continuous scale and as categorical variables, and preterm birth before 34 weeks and delivery within 7 days after admission as outcomes. Models were adjusted for treatment allocation. An interaction term between CL2 and category of Δ_CL_ was tested and, in the case of a significant interaction, was added to the model. Predicted probabilities and their corresponding 95% CIs were calculated for the risk of preterm birth before 34 weeks and delivery within 7 days after admission and plotted against CL2. A reference line corresponding to a probability of 10% was added, as that is the approximate baseline risk of preterm birth in women after an episode of threatened preterm labor^19^.

Lastly, a sensitivity analysis was conducted including only women randomized to receive no intervention in the original study, as the original trial suggested a non‐significant trend towards an increased risk of preterm birth before 34 weeks in the pessary group^14^.

All outcomes were reported as adjusted odds ratios (aOR) with 95% CIs. *P* < 0.05 was considered to indicate statistical significance. Data were analyzed using IBM SPSS Statistics version 26 (IBM Corp., Armonk, NY, USA) and R version 4.0.2 (R Foundation for Statistical Computing, Vienna, Austria; https://www.R‐project.org/).

## RESULTS

**Figure 1:**
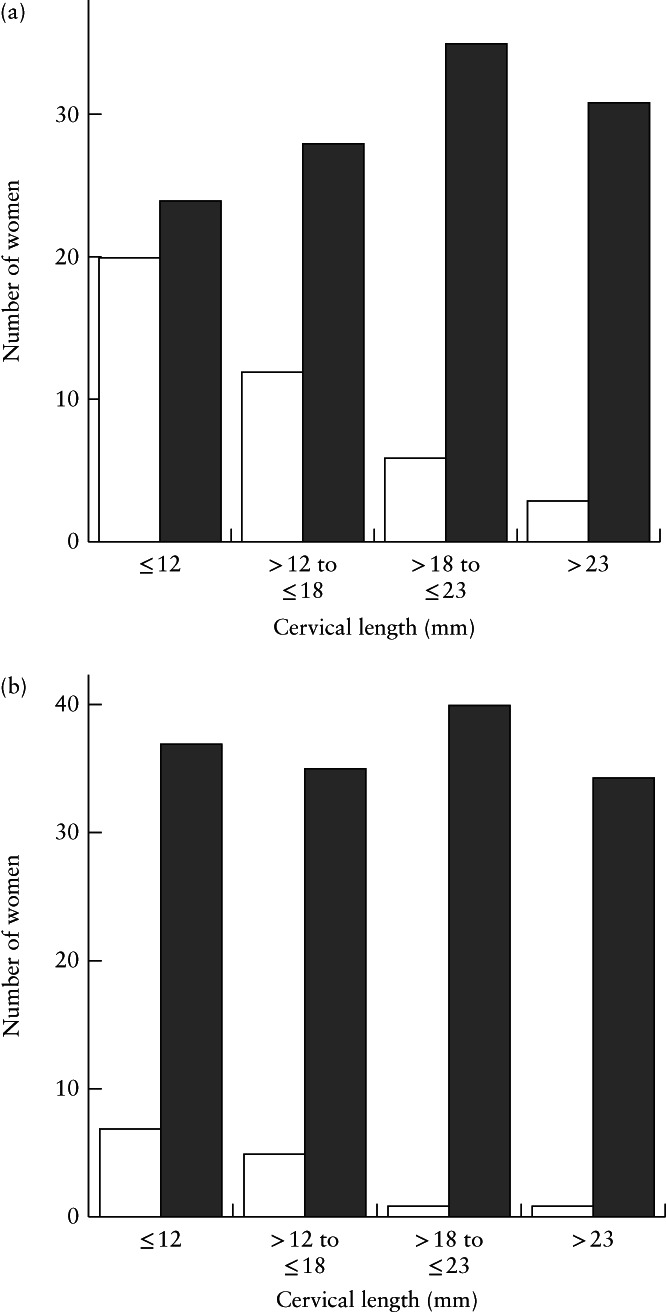
Numbers of women with preterm birth before 34 weeks (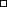) *vs* at or after 34 weeks (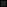) (a) and numbers with delivery within 7 days (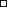) *vs* after 7 days (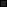) following admission for threatened preterm labor (b), according to cervical length measured at least 48 h after admission.

In total, 164 women were analyzed in the study, of whom 129 had a singleton pregnancy and 82 were randomized to receive a cervical pessary. Table 1 shows their baseline characteristics. Table 2 shows the rate of preterm birth for the three categories of Δ_CL_. Overall, 8.5% (*n* = 14) of women delivered within 7 days after admission for threatened preterm labor and 25.0% (*n* = 41) delivered before 34 weeks. The number of women with preterm birth before 34 weeks and delivery within 7 days after admission is presented against quartiles of CL2 in Figure 1. The incidence of both outcomes decreased with higher quartiles of CL2 (Figure 1).

**Table 1:** Baseline characteristics of 164 women included in the study

Characteristic	Value
Age (years)	30.1 ± 4.9
BMI ($\mathrm{kg}/m^{2}$)	23.0 (20.5–25.9)
Type of gestation	
Singleton	129 (78.7)
Twin	35 (21.3)
Ethnicity	
Caucasian	113/140 (80.7)
Non‐Caucasian	27/140 (19.3)
Higher education	40/73 (54.8)
Smoker	22/141 (15.6)
Obstetric history	
Nulliparous	96 (58.5)
Parous without history of PTB	33 (20.1)
Parous with history of PTB	35 (21.3)
Other risk factors for PTB*	41/122 (33.6)
Use of ART	20 (12.2)
Preventive use of progesterone†	13/35 (37.1)
Gestational age at randomization (weeks)	28.7 (27.0–30.4)
Cervical length at admission (mm)	17.0 (12.0–22.0)
Cervical length at least 48 h after admission (mm)‡	18.0 (12.0–23.0)
Change in cervical length (mm)	0.0 (–2.0 to 4.0)

Data are presented as mean ± SD, median (interquartile range), *n* (%) or *n*/*N* (%).

* Other risk factors included large loop excision of transformation zone or conization, uterine anomaly, uterine surgery, history of cerclage and family history of preterm birth (PTB).

† In women with a history of PTB.

‡ Measured after contractions had ceased.

ART, assisted reproductive technology; BMI, body mass index.

**Table 2:** Rate of preterm birth (PTB) according to category of change in cervical length (Δ_CL_) between admission for threatened preterm labor and at least 48 h later when contractions had ceased

Timing of PTB	Category of Δ_CL_		
	Decrease (Δ_CL_ < −2 mm) (*n* = 34)	No change (Δ_CL_ ≥ −2 mm and ≤ 2 mm) (*n* = 77)	Increase (Δ_CL_ > 2 mm) (*n* = 53)
Within 7 days after admission	4 (11.8)	10 (13.0)	0 (0)
Before 34 weeks	13 (38.2)	23 (29.9)	5 (9.4)

Data are given as *n* (%).

**Figure 2:**
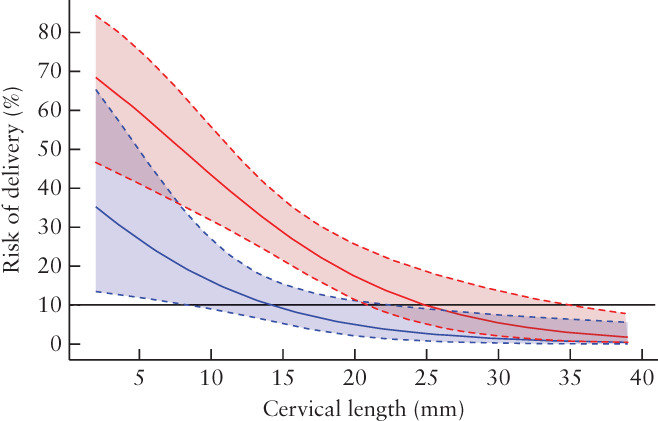
Predicted risk, with 95% CI, of preterm birth before 34 weeks (red) and delivery within 7 days after admission for threatened preterm labor (blue), according to cervical length measured at least 48 h after admission. 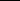, Reference line at 10% predicted risk.

As shown in Table 3, greater CL2 was associated with a lower risk of preterm birth before 34 weeks (aOR, 0.90

(95% CI, 0.84–0.96)) and of delivery within 7 days after admission (aOR, 0.91 (95% CI, 0.82–1.02)). In Figure 2, predicted risks for the two outcomes are displayed against CL2. CL2 above 25 mm and 15 mm corresponded to a predicted risk of 10% or less for preterm birth before 34 weeks and delivery within 7 days after admission, respectively (Figure 2).

**Table 3:** Association of preterm birth (PTB) with cervical length at least 48 h after admission for threatened preterm labor (CL2) and with change in cervical length between admission and CL2 (Δ_CL_)

Parameter	PTB before 34 weeks				PTB within < 7 days after admission			
	OR (95% CI)	*P*	aOR (95% CI)	*P*	OR (95% CI)	*P*	aOR (95% CI)	*P*
CL2 (mm)	0.90 (0.84–0.96)	0.002	0.90 (0.84–0.96)	0.003	0.91 (0.81–1.01)	0.07	0.91 (0.82–1.02)	0.09
Δ_CL_ (mm)	0.90 (0.83–0.96)	0.001	0.90 (0.83–0.96)	0.002	0.88 (0.79–0.97)	0.01	0.88 (0.79–0.97)	0.01
Category of Δ_CL_								
Decrease* (Δ_CL_ < −2 mm)	1.43 (0.61–3.33)	0.41	1.45 (0.62–3.41)	0.40	0.88 (0.26–3.03)	0.84	0.89 (0.26–3.07)	0.85
Increase* (Δ_CL_ > 2 mm)	0.24 (0.09–0.68)	0.01	0.24 (0.09–0.69)	0.01	NA†		NA†	

* Using the group with no change in cervical length as reference.

† Odds ratios (OR) could not be estimated, as none of the women with an increase in cervical length delivered within 7 days after admission.

aOR, odds ratios adjusted for treatment allocation; NA, not available.

**Figure 3:**
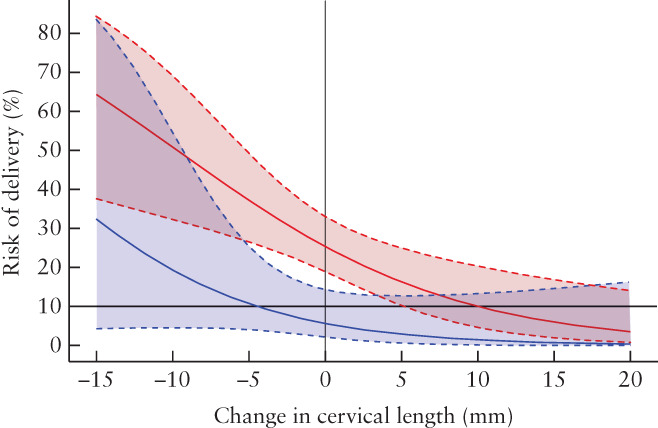
Predicted risk, with 95% CI, of preterm birth before 34 weeks (red) and delivery within 7 days after admission for threatened preterm labor (blue), according to change in cervical length between admission and at least 48 h later. 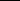, Reference line at 10% predicted risk.

Women with an increase in cervical length between admission and at least 48 h after arrested preterm labor had the lowest rates of preterm birth within 7 days after admission and before 34 weeks (Table 2). Table 3 also illustrates that, as Δ_CL_ increased on a continuous scale, the risk of preterm birth before 34 weeks (aOR, 0.90 (95% CI, 0.83–0.96)) and delivery within 7 days (aOR, 0.88 (95% CI, 0.79–0.97)) decreased. This is illustrated graphically in Figure 3. Table 3 shows a similar trend for Δ_CL_ as a categorical variable, as women with an increase in cervical length between the two timepoints had a lower risk of preterm birth before 34 weeks compared to those with no change in cervical length (aOR, 0.24 (95% CI, 0.09–0.69)). The aOR for delivery within 7 days following admission could not be estimated, as there were no women with this outcome and an increase in cervical length. A decrease in cervical length between CL1 and CL2, when compared with no change in cervical length, was not associated with an increased risk for preterm birth before 34 weeks (aOR, 1.45 (95% CI, 0.62–3.41)) or delivery within 7 days after admission (aOR, 0.89 (95% CI, 0.26–3.07)).

**Figure 4:**
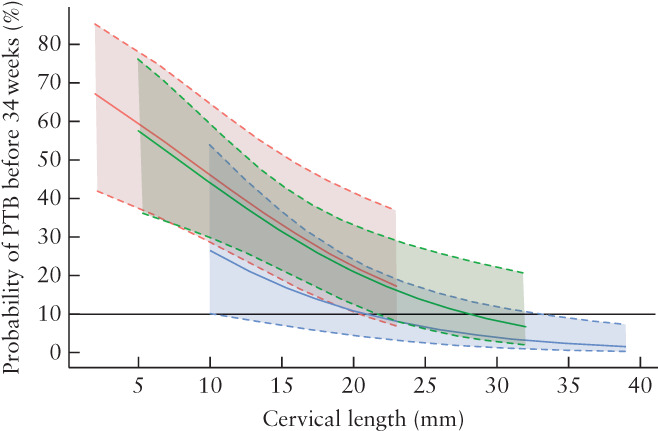
Predicted risk, with 95% CI, of preterm birth (PTB) before 34 weeks, according to cervical length measured at least 48 h after admission for threatened preterm labor and category of change in cervical length between admission and at least 48 h later (increase in cervical length (blue), decrease in cervical length (red) and no change in cervical length (green)). 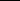, Reference line at 10% predicted risk.

In Figure 4, CL2 and Δ_CL_ as a categorical variable were combined, as there was no significant interaction between the two variables (*P* = 0.52). Figure 4 shows that, for any value of CL2, the predicted risk of preterm birth before 34 weeks was lower in women with an increase in cervical length between CL1 and CL2 compared to women with a decrease or no change in cervical length between the two timepoints.

Sensitivity analysis in women randomized to receive no intervention did not identify any major differences compared with the overall analysis (Tables S1 and S2, and Figures [Link], [Link]).

## DISCUSSION

The aim of this *post‐hoc* analysis was to determine the association between cervical length measured 48 h after arrested preterm labor, expressed as both a single value and a change relative to the measurement at admission, and preterm birth in high‐risk women. The main findings of this study suggest that women whose cervical length increases between admission for threatened preterm labor and 48 h later have a significantly lower risk of preterm birth before 34 weeks than do women with no change in cervical length.

### Strengths and limitations

The present study has a number of important strengths. It is one of the first studies to investigate the association between cervical length after arrested preterm labor and preterm birth in high‐risk women. Moreover, there were no missing data for the cervical length measurements, which reduced the risk of selection bias. However, there are also several limitations that must be considered. First of all, this research was a *post‐hoc* analysis of a RCT, in which half of the women received a cervical pessary. Although the original trial found no significant effect of the pessary on any of the outcome measures, a trend towards an increased risk of preterm birth was found^14^. To account for this, treatment allocation was included as a covariate in our analysis, and a sensitivity analysis was performed, showing robust results. However, this came at the expense of statistical power, as half of the population was not analyzed.

Second, a power calculation was not performed for this *post‐hoc* analysis. Therefore, caution should be used when interpreting the results from this analysis, especially for groups with a small number of events. Another limitation of the study is the possible influence of regression to the mean on the second cervical‐length measurement.

As the first measurement was performed at admission for threatened preterm labor when women were having contractions, a higher second measurement after 48 h when contractions have ceased is more likely. However, taking into account the margin of error used in this study, it is probable that the majority of such cases fell into the group of no change in cervical length, and that the categories of increase and decrease in cervical length between admission and at least 48 h later thus reflect a true effect of change. This is supported by the fact that change in cervical length between the two timepoints (Δ_CL_) had a normal distribution, with a mean of 1 mm, which is within the margin of error. A final limitation is that, in The Netherlands, women with an increased risk of preterm birth are identified with a selection algorithm based on both cervical length and fetal fibronectin status, while in other countries this selection is done based on either cervical length or fetal fibronectin. This may limit the implementation of cervical‐length measurement after arrested preterm labor in centers with no combined screening algorithm owing to differences in the *a‐priori* risk of preterm birth.

### Comparison with previous literature

There have been a limited number of studies assessing cervical length up to 48 h after threatened preterm labor, but previous research that investigated the association between cervical length and the risk of preterm birth in women with threatened preterm labor also reported an inverse relationship^12^, ^20^, ^21^, ^22^, ^23^, ^24^. Although previous studies reported a cut‐off of 15 mm, our analysis of cervical length as a continuous variable resulted in a higher cut‐off of 25 mm^12^, ^25^, ^26^. This discrepancy may be due to the higher *a‐priori* risk of preterm birth in our study population, suggesting that a higher cut‐off point should be applied to this group.

A possible explanation for lengthening of the cervix after 48 h is the effect of tocolysis. As suggested by Rozenberg *et al*.^20^, the cervix may lengthen temporarily after 48 h as a result of relaxation of the myometrium. Since all women in the present study received tocolysis, it is possible that an increase in cervical length after 48 h occurred in those women in whom tocolysis was most successful, leading to lower rates of preterm birth in this group. However, although Rozenberg *et al*. did not find an association between lengthening of the cervix after 48 h and preterm birth, our study did detect a significant effect. This may be explained by differences in the study populations, as our study included only women with an *a‐priori* high risk of preterm birth. This was also reflected by the rate of preterm birth, which was almost 10% higher than in the study of Rozenberg *et al*.^20^.

The findings of this study show that a decrease in cervical length increases the risk of preterm birth before 34 weeks' gestation, which is consistent with previous research^27^, ^28^, ^29^, ^30^, ^31^. This finding was not statistically significant, which may be because of the small number of women whose cervical length decreased (*n* = 34), resulting in low statistical power.

Almost 9% of the study population delivered within 7 days after admission for threatened preterm labor, which is similar to the rates reported previously^22^. None of the women with an increase in cervical length delivered within 7 days after admission. However, as the number of women who delivered within 7 days was relatively small (*n* = 14), this finding, as well as the graphical extrapolations, should be interpreted with caution. Other studies found cervical length to be predictive of delivery within 7 days, with several studies establishing 15 mm as a relevant cut‐off^22^, ^26^, ^32^, ^33^, ^34^. This cut‐off was also found in the current study.

### Conclusions

The findings of this study suggest that pregnant women with an increase in cervical length between admission for threatened preterm labor and cessation of contractions at least 48 h later have a low risk of preterm birth before 34 weeks.

Further research should focus on improving the primary algorithms for risk stratification of preterm birth (prediction models) in women with threatened preterm labor. This could be achieved in large cohort studies and should incorporate measurements in women who do not deliver preterm in order to develop secondary algorithms for risk stratification to quantify the remaining risk of preterm birth more precisely.

## Supporting information


**Figure S1** Predicted risk, with 95% CIs, of preterm birth before 34 weeks (red) and delivery within 7 days after admission for threatened preterm labor (blue), according to cervical length at least 48 h after admission, in women randomized to no intervention. Reference line at 10% predicted risk (black, dashed line) is shown.

**Figure S2** Predicted risk, with 95% CIs, of preterm birth before 34 weeks (red) and delivery within 7 days after admission for threatened preterm labor (blue), according to change in cervical length between admission and at least 48 h later, in women randomized to no intervention. Reference line at 10% predicted risk (black, dashed line) is shown.

**Figure S3** Predicted risk, with 95% CIs, of preterm birth before 34 weeks, according to cervical length at least 48 h after admission for threatened preterm labor and category of change in cervical length between admission and at least 48 h later, in women randomized to no intervention. Reference line at 10% predicted risk (black, dashed line) is shown.

**Table S1** Rate of preterm birth (PTB) in women randomized to no intervention, according to category of change in cervical length (Δ_CL_) between admission for threatened preterm labor (CL1) and at least 48 h later (CL2)

**Table S2** Association of preterm birth (PTB) with cervical length at least 48 h after admission for threatened preterm labor (CL2) and change in cervical length (Δ_CL_) between admission (CL1) and CL2 in women randomized to no intervention

## Data Availability

The data that support the findings of this study are available on request from the corresponding author. The data are not publicly available due to privacy or ethical restrictions.
