# Supplementary figures and images for: Change in cervical length after arrested preterm labor and risk of preterm birth
Source: Ultrasound Obstet Gynecol. 2021 Nov 1;58(5):750–6. doi: 10.1002/uog.23653 (PMC8596619; doi:10.1002/uog.23653)

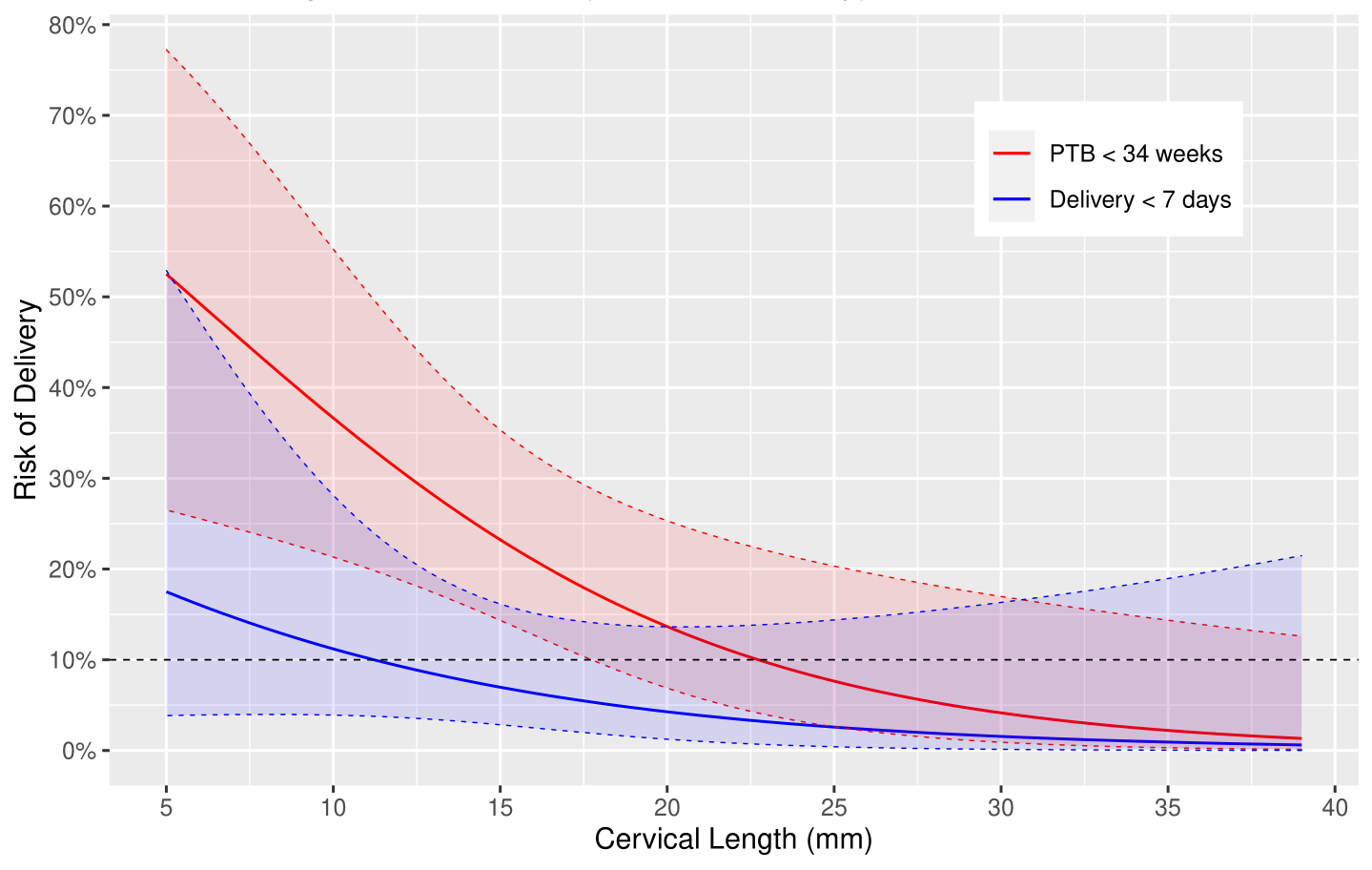

Supplement: Supplementary file 1 — Figure S1 Predicted risk, with 95% CIs, of preterm birth before 34 weeks (red) and delivery within 7 days after admission for threatened preterm labor (blue), according to cervical length at least 48 h after admission, in women randomized to no intervention. Reference line at 10% predicted risk (black, dashed line) is shown. [file UOG-58-750-s001.tiff]

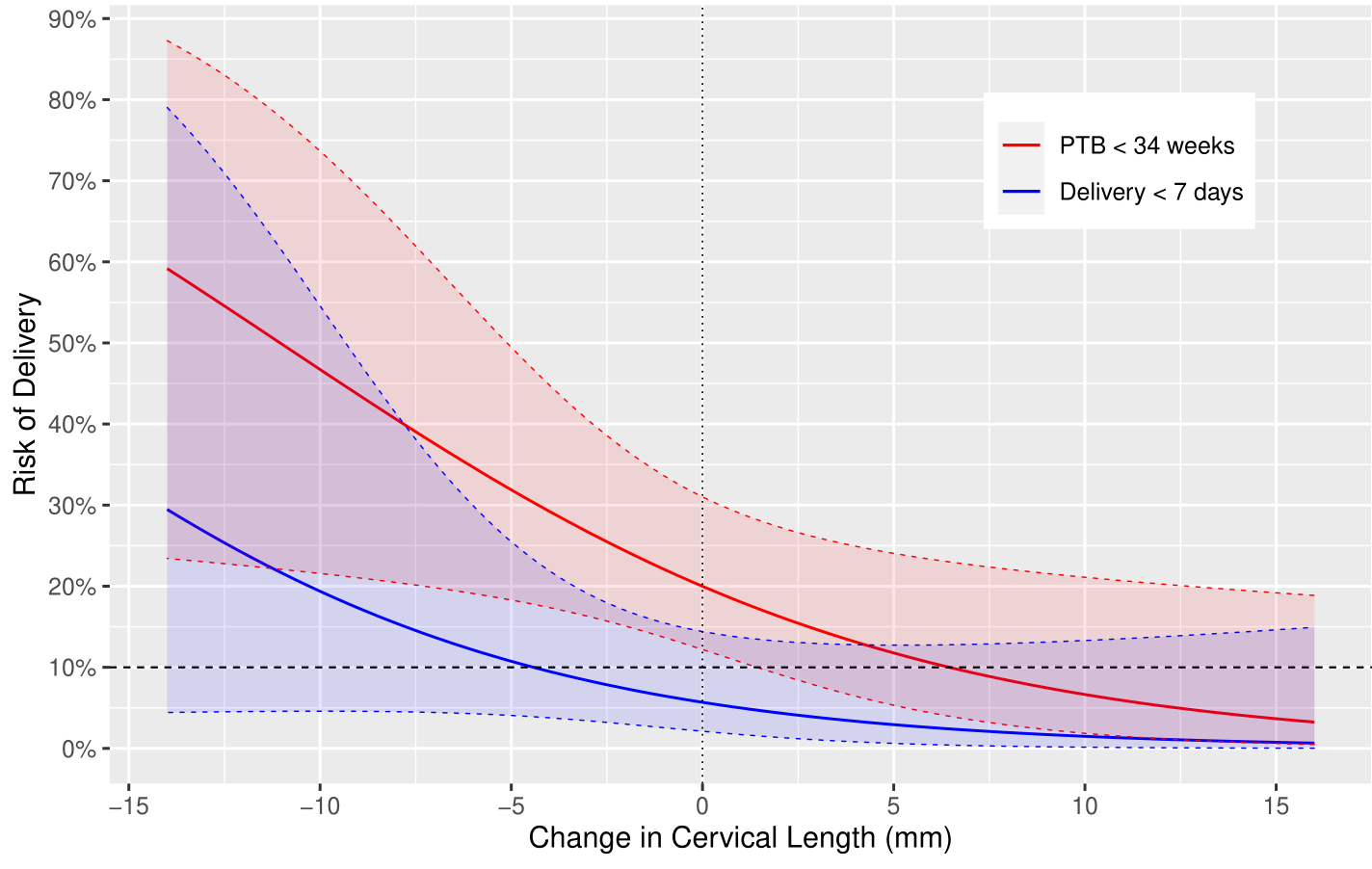

Supplement: Supplementary file 2 — Figure S2 Predicted risk, with 95% CIs, of preterm birth before 34 weeks (red) and delivery within 7 days after admission for threatened preterm labor (blue), according to change in cervical length between admission and at least 48 h later, in women randomized to no intervention. Reference line at 10% predicted risk (black, dashed line) is shown. [file UOG-58-750-s005.tiff]

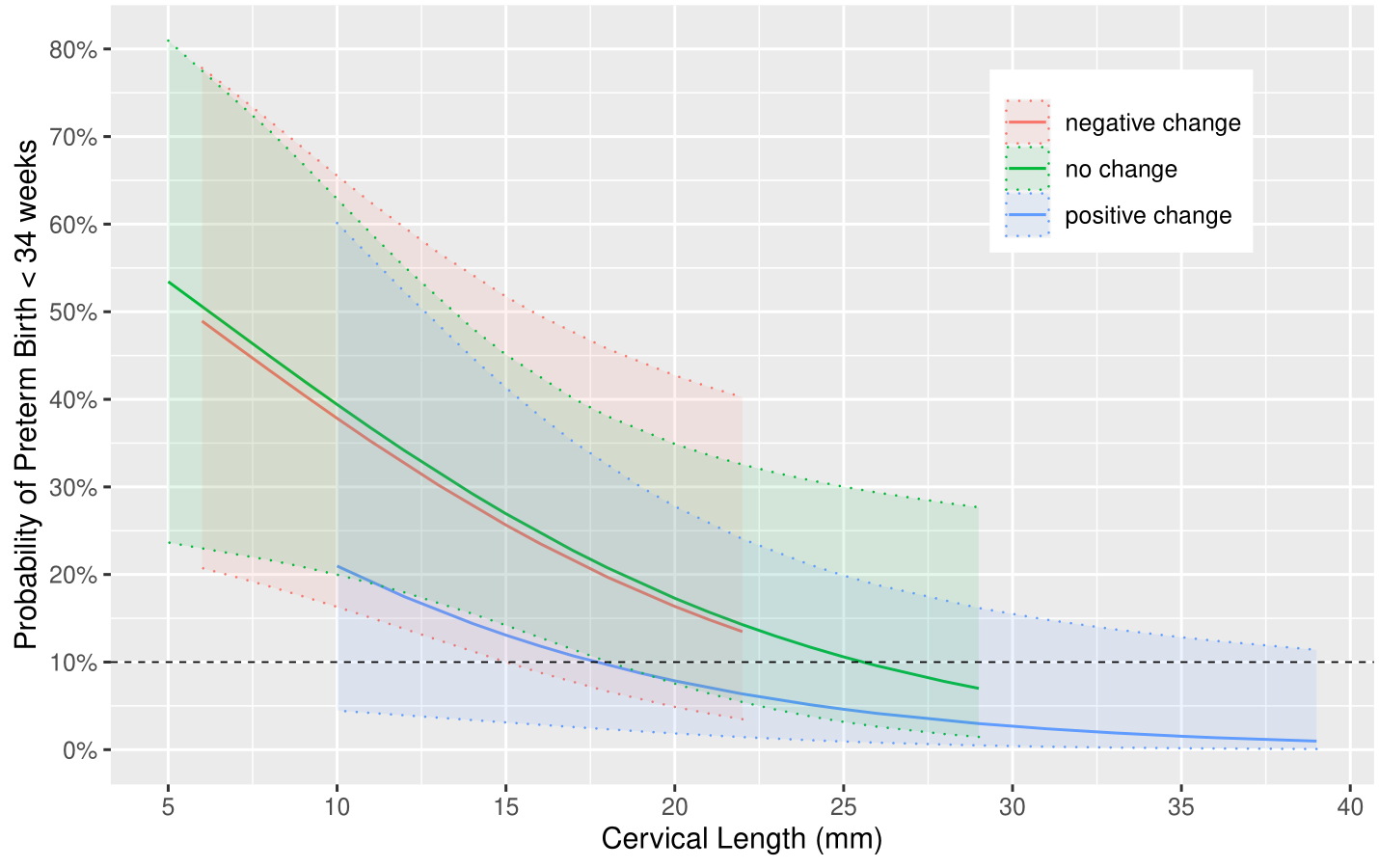

Supplement: Supplementary file 3 — Figure S3 Predicted risk, with 95% CIs, of preterm birth before 34 weeks, according to cervical length at least 48 h after admission for threatened preterm labor and category of change in cervical length between admission and at least 48 h later, in women randomized to no intervention. Reference line at 10% predicted risk (black, dashed line) is shown. [file UOG-58-750-s002.tiff]
