# Supplementary material for: Change in cervical length after arrested preterm labor and risk of preterm birth
Source: Ultrasound Obstet Gynecol. 2021 Nov 1;58(5):750–6. doi: 10.1002/uog.23653 (PMC8596619; doi:10.1002/uog.23653)
Supplement: Supplementary file 4 — Table S1 Rate of preterm birth (PTB) in women randomized to no intervention, according to category of change in cervical length (ΔCL) between admission for threatened preterm labor (CL1) and at least 48 h later (CL2) [file UOG-58-750-s004.docx]

**Table S1** Rate of preterm birth (PTB) in women randomized to no intervention, according to category of change in cervical length (Δ_CL_) between admission for threatened preterm labor (CL1) and at least 48 h later (CL2)

| Change in cervical length (Δc=C2-C1) | Frequency (*n*) | PTB <7 days (*n*=14), *n* (%) | PTB <34 weeks (*n*=41), *n* (%) |
| --- | --- | --- | --- |
| Decrease  (Δc < -2 mm) | 17 | 1 (5.9) | 5 (29.4) |
| No change  (Δc ≥-2 mm & Δc ≤2 mm) | 37 | 4 (10.8) | 9 (24.3) |
| Increase  (Δc > 2mm) | 28 | 0 | 2 (7.1) |
