# Supplementary material for: Change in cervical length after arrested preterm labor and risk of preterm birth
Source: Ultrasound Obstet Gynecol. 2021 Nov 1;58(5):750–6. doi: 10.1002/uog.23653 (PMC8596619; doi:10.1002/uog.23653)
Supplement: Supplementary file 5 — Table S2 Association of preterm birth (PTB) with cervical length at least 48 h after admission for threatened preterm labor (CL2) and change in cervical length (ΔCL) between admission (CL1) and CL2 in women randomized to no intervention [file UOG-58-750-s003.docx]

**Table S2**  Association of preterm birth (PTB) with cervical length at least 48 h after admission for threatened preterm labor (CL2) and change in cervical length (Δ_CL_) between admission (CL1) and CL2 in women randomized to no intervention

|  | PTB <34 weeks | | | Birth <7 days | | |
| --- | --- | --- | --- | --- | --- | --- |
|  | **OR** | **95% C.I.** | **p** | **OR** | **95% C.I.** | **p** |
| **Cervical length (mm) after at least 48h (continuous)** | 0.89 | 0.81 to 0.99 | 0.03 | 0.92 | 0.78 to 1.08 | 0.32 |
| **Change (C2-C1) in cervical length (mm) (continuous)** | 0.88 | 0.78 to 0.97 | 0.02 | 0.87 | 0.73 to 1.0 | 0.10 |
| **Change (C2-C1) in cervical length (categorical) (reference: no change)** |  |  |  |  |  |  |
| Decrease (Δc < -2 mm) | 1.30 | 0.36 to 4.69 | 0.69 | 0.52 | 0.05 to 5.00 | 0.57 |
| Increase (Δc > 2 mm) | 0.24 | 0.05 to 1.21 | 0.08 |  | N/E* |  |

*N/E: not estimable as none of the women with an increase in cervical length delivered within 7 days of admission
